# Structural Validity of the Arabic Roland–Morris Disability Questionnaire Using Confirmatory Factor Analysis in Patients with Low Back Pain

**DOI:** 10.3390/jcm15124527

**Published:** 2026-06-11

**Authors:** Abdulrahman M. Alsubiheen, Mishal M. Aldaihan, Ali H. Alnahdi

**Affiliations:** Department of Rehabilitation Sciences, College of Applied Medical Sciences, King Saud University, P.O. Box 10219, Riyadh 11433, Saudi Arabiamishaldaihan@ksu.edu.sa (M.M.A.)

**Keywords:** low back pain, RMDQ, structural validity, confirmatory factor analysis, Arabic, PROM

## Abstract

**Background/Objective:** Low back pain (LBP) is a leading cause of disability worldwide, and patient-reported outcome measures such as the Roland–Morris Disability Questionnaire (RMDQ) are essential for assessing LBP-related disability. While the Modern Standard Arabic version of the RMDQ has demonstrated preliminary reliability, its structural validity has not been thoroughly evaluated. This study aimed to assess the structural validity of the Modern Standard Arabic RMDQ using confirmatory factor analysis (CFA). **Methods:** A cross-sectional study was conducted for 113 patients with LBP recruited from outpatient physical therapy clinics in Saudi Arabia. Participants completed the Modern Standard Arabic RMDQ, a 24-item instrument scored dichotomously. CFA was performed using the Weighted Least Squares Mean and Variance adjusted estimator to test a unidimensional model. Model fit was assessed using Root Mean Square Error of Approximation (RMSEA), Standardized Root Mean Square Residual (SRMR), Tucker–Lewis Index (TLI), and Comparative Fit Index (CFI). Reliability was evaluated using McDonald’s omega (ω). **Results:** The initial one-factor CFA model showed close to acceptable fit (RMSEA = 0.044; SRMR = 0.149; TLI = 0.94; CFI = 0.93). After accounting for significant residual correlations between item pairs (items 4 & 21; 13 & 18), model fit improved (Δχ^2^ = 22.33; Δdf = 2; *p* < 0.001) (RMSEA = 0.038; SRMR = 0.145; TLI = 0.95; CFI = 0.95). Most items had significant loadings on the latent construct, except item 2. McDonald’s ω was 0.91, indicating excellent internal consistency. **Conclusions:** The findings of this study provide supportive evidence for the structural validity and internal consistency of the Modern Standard Arabic version of the RMDQ and suggest the presence of a dominant unidimensional structure. The Arabic RMDQ may be useful for assessing LBP-related disability in Arabic-speaking patients with LBP, although further validation studies are warranted.

## 1. Introduction

Low back pain (LBP) stands among the most prevalent musculoskeletal conditions worldwide and is a major contributor to disability and economic burden [1,2]. Its effects are far-reaching, often leading to reduced physical functioning, decreased productivity, and diminished quality of life [3]. To assess the impact of LBP on individuals’ daily lives, healthcare providers and researchers increasingly depend on patient-reported outcome measures (PROMs) [4]. These tools are critical for capturing patients’ subjective experiences related to functional disability and overall health status [5]. As such, PROMs serve a central role in informing clinical decisions, tracking patient progress, and determining the effectiveness of therapeutic interventions.

The Roland–Morris Disability Questionnaire (RMDQ) is among the most extensively utilized PROMs for assessing functional disability due to LBP [6,7]. Developed by Roland and Morris, the RMDQ consists of 24 dichotomously scored items derived from the sickness impact profile, with each item reflecting a specific functional limitation that patients may experience. The original scale [6] was constructed to measure a single dimension of physical disability related to LBP and has demonstrated good reliability and construct validity across a range of populations and languages [8,9,10]. However, the internal structure and dimensionality of the RMDQ have been subjects of ongoing debate. While some studies have supported its unidimensional nature [11,12,13,14,15,16,17], others have identified multidimensional solutions, suggesting potential subdomains [9,18,19,20,21,22]. These inconsistent findings underline the importance of verifying the structural validity of the RMDQ when it is adapted for use in different cultural and linguistic settings [10].

In Arabic-speaking populations, the burden of LBP is substantial [23,24], and the availability of culturally and linguistically adapted PROMs is vital. A Modern Standard Arabic version of the RMDQ has been previously translated and demonstrated preliminary evidence of reliability and construct validity [25]. Nevertheless, evidence regarding its structural validity and the degree to which the scores of an instrument adequately reflect the dimensionality of the construct being measured [26] remains limited. According to the COnsensus-based Standards for the selection of health Measurement INstruments (COSMIN) guidelines, evaluating structural validity through confirmatory factor analysis (CFA) is critical, as it determines whether the theoretical construct structure is empirically supported [27]. Establishing the structural validity of the Arabic RMDQ is a prerequisite for ensuring its meaningful use in outcome evaluation and for making valid clinical inferences based on its scores. Moreover, structural validity is considered one of the most important measurement properties because it forms the basis for the interpretation of questionnaire scores and subsequent validity testing. Without adequate evidence supporting the internal structure of an instrument, conclusions drawn from total scores may be questionable.

Therefore, the objective of this study was to evaluate the structural validity of the Modern Standard Arabic version of the RMDQ in patients with LBP using CFA. We hypothesized that the Arabic RMDQ would demonstrate a unidimensional structure consistent with the original version, and that the model would exhibit acceptable fit indices, supporting its use as a valid measure of LBP-related disability in Arabic-speaking populations.

## 2. Materials and Methods

### 2.1. Study Design

This investigation employed a cross-sectional methodology to assess the structural validity of the Arabic RMDQ among individuals experiencing LBP.

### 2.2. Setting and Participants

This study enrolled participants from outpatient physical therapy clinics located in the central region of Saudi Arabia, specifically at Alrass General Hospital and the Security Forces Hospital. Recruitment was conducted using a convenience sampling method, whereby all eligible individuals who visited the participating clinics between June 2022 and June 2023 were invited to participate. Prior to enrollment, each participant received detailed information about the study’s aims, procedures, and their right to voluntary participation, after which written informed consent was obtained. The study protocol received ethical clearance from King Saud University ethics committee (E-20-5529) and the Security Forces Hospital ethics committee (22-601-37). Participant recruitment at Alrass General Hospital was conducted under the ethical approval and collaborative research agreements associated with the study protocol approved by the participating institutional review boards.

Participants were eligible for inclusion if they: (1) were aged 18 years or older; (2) had been referred to physical therapy for the management of LBP; and (3) demonstrated adequate Arabic literacy to independently complete the study questionnaires. Eligible individuals had been previously diagnosed by consultant physicians in orthopedic, spine, or primary healthcare settings before being referred to physical therapy. Exclusion criteria included: (1) the presence of systemic illnesses; (2) cardiopulmonary or neurological disorders that impaired functional capacity; (3) musculoskeletal conditions unrelated to LBP causing functional limitations as reported by the patients; and (4) a history of spinal surgery.

### 2.3. Procedure

During the participants’ first visit to the physical therapy clinic, eligibility was confirmed and written informed consent was obtained. Following this, participants completed the paper-based version of the RMDQ on-site. In addition, demographic and clinical data relevant to each participant were collected and documented at that time.

### 2.4. Outcome Measures

#### Roland–Morris Disability Questionnaire (RMDQ)

The RMDQ was used to assess self-reported functional disability related to LBP. It consists of 24 items that describe limitations in daily activities commonly affected by LBP [6,7]. Participants were instructed to respond with “yes” or “no” to each item, indicating whether the statement applied to them on the day of assessment. Each “yes” response is scored as 1, resulting in a total score ranging from 0 to 24, with higher scores reflecting greater disability. In this study, the validated Arabic version of the RMDQ was administered. This version has demonstrated adequate internal consistency (Cronbach’s α = 0.73) and excellent test–retest reliability (intraclass correlation coefficient [ICC] = 0.90) [25] in Arabic-speaking populations but without prior evidence regarding its structural validity.

### 2.5. Statistical Analysis

#### Confirmatory Factor Analysis (CFA)

To examine the structural validity of the Arabic RMDQ, a CFA was conducted. The hypothesized model consisted of a single latent variable, back-related disability, underlying 24 observed indicators corresponding to the RMDQ items. Prior to analysis, data were screened for distributional characteristics. Most items demonstrated skewed univariate distributions, and multivariate normality was not met, as evidenced by significant skewness and kurtosis in Mardia’s test (*p* < 0.001). Due to the ordinal nature of the RMDQ items and the violation of multivariate normality assumptions, the analysis was conducted using the Weighted Least Squares Mean and Variance adjusted (WLSMV) estimator. This robust estimation method is well-suited for CFA models involving ordinal data, as it does not assume normally distributed variables and provides corrected standard errors and fit statistics [28,29,30,31]. Tetrachoric correlation matrices were used in the CFA because the RMDQ items are dichotomous in nature and are assumed to reflect underlying continuous latent response variables. Standardized factor loadings were examined to evaluate the contribution of each item to the latent construct, with higher loadings indicating stronger relationships with back-related disability. Items with low standardized loadings were carefully evaluated in relation to their theoretical relevance and contribution to the overall model.

Model fit was assessed using a range of indices: chi-square statistics (χ^2^), the Root Mean Square Error of Approximation (RMSEA), the Standardized Root Mean Square Residual (SRMR), the Tucker–Lewis Index (TLI), and the Comparative Fit Index (CFI). A model was considered to have acceptable fit based on the following criteria: RMSEA ≤ 0.06, SRMR ≤ 0.08, TLI and CFI values ≥ 0.95 [27,32,33]. Fit indices were scaled to reflect the categorical nature of the data. To further explore potential areas of misfit, standardized residuals and modification indices were assessed [30,34]. The Arabic RMDQ reliability (internal consistency) was assessed using McDonald’s ω [35,36]. All statistical procedures were carried out using JASP software (version 0.19.3) [37] and Jamovi (Version 2.6.26) [38].

### 2.6. Sample Size Estimation

The required sample size to conduct CFA of the RMDQ was determined utilizing the semPower.aPriori function within the semPower R package (version 2.1.3) [39]. The analysis was based on detecting model misspecification in a CFA model with 252 degrees of freedom using an RMSEA-based approach with an RMSEA effect size of 0.06. Assuming a null RMSEA of 0.00, an alternative RMSEA of 0.06, an alpha level of 0.05, a desired statistical power of 0.80, and Diagonally Weighted Least Squares (DWLS) as the estimation method, the minimum required sample size was estimated to be 69 participants.

## 3. Results

A total of 113 individuals with LBP were enrolled in the study. As presented in Table 1, the sample primarily consisted of middle-aged adults with no difference in the proportion of male and female participants (*p* = 0.71). Most of the participants presented with chronic symptoms.

To evaluate the structural validity of the Arabic NDI, a CFA was performed specifying a single latent factor representing low-back-related disability, measured by 24 observed RMDQ items. None of the participants had missing responses to any of the RMDQ items. The initial CFA model yielded the following fit statistics: χ^2^(252) = 305.79, *p* = 0.012; RMSEA = 0.044 (90% CI: 0.022–0.060); SRMR = 0.149; TLI = 0.94; and CFI = 0.93. While not ideal, the overall pattern of fit indices indicated that the unidimensional model approached acceptable fit thresholds.

**Figure 1:**
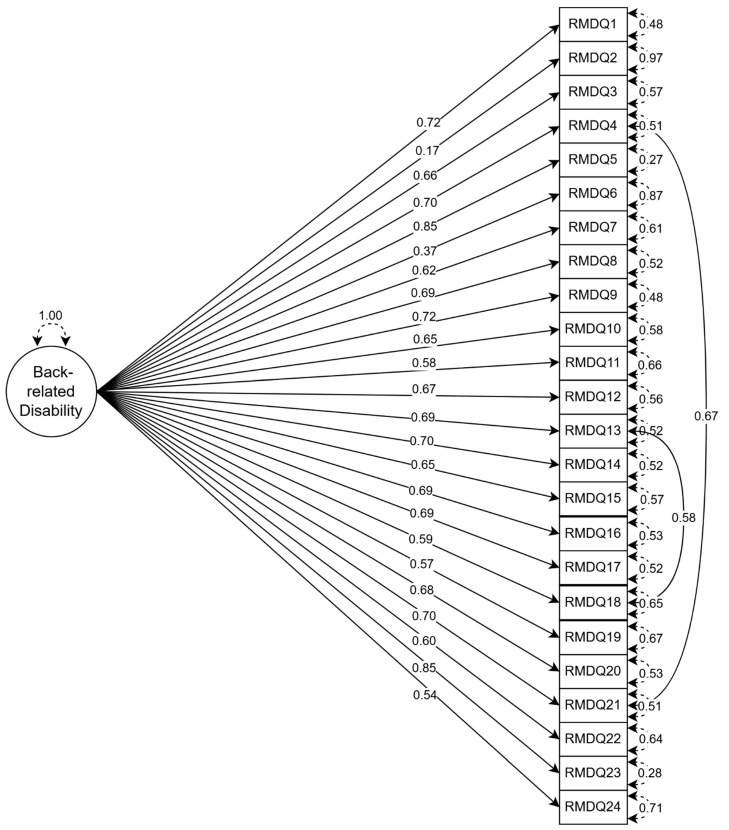
The Roland–Morris Disability Questionnaire (RMDQ) confirmatory factor analysis model included a single latent factor representing back-related disability. A correlated error term was specified between item 4 and item 21 and between item 13 and item 18. Residual variances of the items were depicted using curved dotted double-headed arrows.

Inspection of modification indices highlighted a substantial residual correlation between item 4 (“Because of my back I am not doing any of the jobs that I usually do around the house”) and item 21 (“I avoid heavy jobs around the house because of my back”), and also between item 13 (“My back is painful almost all the time”) and item 18 (“I sleep less well because of my back”). Covariance paths were added between the error terms of these two item pairs. These correlated residuals were introduced post hoc based on both statistical evidence and conceptual overlap between item contents. The revised model significantly improved the fit of the model (Δχ^2^ = 22.33; Δdf = 2; *p* < 0.001) and demonstrated an improved fit to the data: χ^2^(250) = 289.68, *p* = 0.043; RMSEA = 0.038 (90% CI: 0.007–0.055); SRMR = 0.145; TLI = 0.95; and CFI = 0.95. These results suggest that the modified unidimensional model provides supportive evidence for a dominant unidimensional structure of the Arabic RMDQ, although some residual model misfit remained, as reflected by the elevated SRMR value in particular (Figure 1).

Standardized factor loadings for most of the RMDQ items indicated significant positive and moderate to strong associations with the underlying construct of low-back-related disability, with loadings ranging from 0.37 to 0.85 (Table 2; Figure 1). Only item 2 (“I change position frequently to try and get my back comfortable”) showed a non-significant low association with the underlying construct of low-back-related disability with a standardized loading of 0.17 (Table 2; Figure 1). The residual correlation between item 4 and item 21; item 13 and 18 demonstrated significant positive moderate associations (0.67, 95% CI: 0.41–0.93; *p* < 0.001) (0.58, 95% CI: 0.31–0.86; *p* < 0.001), supporting the decision to include the correlated error terms in the model. The Arabic RMDQ showed excellent reliability and internal consistency with McDonald’s ω value of 0.91. Given the weak and non-significant loading of item 2, an exploratory sensitivity analysis was conducted, excluding this item. The revised 23-item model demonstrated similar internal consistency (McDonald’s ω = 0.91) with improved fit indices: χ^2^(228) = 254.30, *p* = 0.112; RMSEA = 0.032 (90% CI: 0.000–0.052); TLI = 0.97; and CFI = 0.97, although SRMR remained elevated at 0.136. These findings suggest that the removal of item 2 improved the model fit but residual misfit is still present despite removing item 2.

Threshold estimates for the dichotomous RMDQ items ranged from −0.829 to 2.104 (Table 2), indicating variability in the level of latent disability required for item endorsement. Items with lower or negative threshold values were more readily endorsed by participants with lower levels of disability, whereas items with higher positive thresholds required greater levels of disability for endorsement. Specifically, item 2 (“I change position frequently to try and get my back comfortable”) demonstrated the lowest threshold (−0.829), suggesting that it represented a relatively mild or commonly experienced limitation. In contrast, item 19 (“Because of my back pain, I get dressed with help from someone else”) showed the highest threshold (2.104), indicating that endorsement of this item required substantially higher levels of disability and therefore reflected a more severe functional limitation. Overall, the distribution of threshold estimates suggests that the RMDQ items capture a broad range of disability severity among individuals with low back pain.

## 4. Discussion

The current investigation sought to assess the structural validity of the Arabic RMDQ through CFA within a cohort of individuals experiencing LBP. The principal inquiry of our research was centered on determining whether the Arabic RMDQ exhibits a unidimensional construct that aligns with the original framework established by Roland and Morris [6]. The CFA outcomes provided supportive evidence for the structural validity of the Arabic version of the RMDQ. The findings provide supportive evidence for a dominant unidimensional structure after the consideration of correlated residuals between two item pairs (items 4 & 21 and 13 & 18), resulting in satisfactory indices of model fit. Furthermore, the Arabic RMDQ demonstrated excellent internal consistency, evidenced by McDonald’s ω coefficient of 0.91. These results suggest that the Arabic RMDQ possesses acceptable structural validity and can be reliably used to assess LBP-related disability within Arabic-speaking populations.

The initial unidimensional CFA model of the Arabic RMDQ demonstrated suboptimal fit, as indicated by the fit indices (RMSEA, TLI, and CFI). However, modification indices revealed significant residual correlations between specific item pairs, and adjusting for these correlations led to a significantly improved model, with TLI and CFI reaching thresholds for acceptable fit (TLI = 0.95; CFI = 0.95). Although RMSEA, CFI, and TLI supported the modified unidimensional model, the SRMR remained elevated above the conventional threshold. This finding suggests the presence of residual model misfit not fully accounted for by the specified model. The elevated SRMR may be partly related to the dichotomous nature of the RMDQ items, sparse endorsement frequencies for some items, residual local dependence between conceptually overlapping items, and the relatively modest sample size for CFA involving categorical indicators. Therefore, the present findings should be interpreted as supportive rather than definitive evidence of unidimensionality. The finding of a unidimensional structure aligns with the original purpose of the RMDQ and supports number of prior validation studies in various cultural settings that have also found a single-factor solution using the CFA approach. Using Dutch and Brazilian versions, Chaiarotto and associates and Pontes-Silva and associates reported the RMDQ to be a unidimensional measure using CFA with a suitable estimation method (diagonally weighted least squares) to account for the ordinal nature of the items and the presence of only two response options [13,40]. Arovah and associates examined the underlying structure of the Indonesian RMDQ using CFA in patients with LBP [17]. The authors reported that the scale has sufficient unidimensionality. Additionally, Jenks and associates suggested sufficient unidimensionality of the RMDQ in patients with LBP [9]. In Brazilian patients with LBP, Frota and associates reported a generally acceptable fit of the unidimensional structure of the RMDQ, but reported a better fit of a unidimensional reduced version of the RMDQ with 15 items [12].

Nonetheless, other studies have used CFA and reported multidimensional structures, suggesting the existence of subdomains within the RMDQ. For instance, Takara and associates proposed a two-factor structure for the RMDQ in community-dwelling older adults, comprising functional capacity and mobility dimensions [22]. It is important to note that the authors did not assess the fit of the common one-factor structure of the RMDQ. Using an Albanian version, Lena and associates reported the RMDQ to have two underlying factors without providing information on how these two factors were determined and without details on the CFA estimation method used [41]. Chala and associates reported the Amharic version of the RMDQ to have inadequate fit to the unidimensional model and reported good fit to a four-factor solution [18]. The authors did not present the fit results for the unidimensional model and provided no information related to the CFA estimation method used. The discrepancy between the findings of these studies and those of the present study may be attributed to differences in the studied populations, such as older adults versus general adult populations. In addition, variations in the estimation methods used across studies may have contributed to these discrepancies, particularly when the ordinal nature of the RMDQ items was not adequately accounted for during the CFA procedures in these studies.

In our study, the standardized factor loadings were moderate or strong for most items, ranging from 0.37 to 0.85, with the exception of item 2 “I change position frequently to try and get my back comfortable”, which demonstrated a weak and statistically non-significant loading (0.17). This finding is noteworthy as it indicates that, unlike the other items, item 2 contributes minimally to the underlying latent construct of disability as defined within the RMDQ. Similar concerns have been highlighted in the previous literature using CFA [12], and also in studies that subjected the RMDQ to the unidimensional Rasch measurement model [14,15,16]. A plausible explanation is that item 2 may capture a behavioral strategy or coping mechanism that patients employ to alleviate discomfort, rather than directly reflecting functional disability. In this sense, the behavior of “changing position” could represent an adaptive response to pain rather than an indicator of limitation or impairment. Consequently, its weak loading raises questions regarding the conceptual alignment of the item with the intended construct of disability. This issue warrants further investigation, particularly in the context of cross-cultural adaptation, as the interpretation and relevance of such behaviors may differ between populations. Moreover, the persistence of this finding across studies suggests that item 2 could be a candidate for closer scrutiny in future validation studies, potentially requiring revision, contextual rewording, or even removal if it continues to show weak psychometric performance. The improved fit observed after excluding item 2 further supports concerns regarding the psychometric performance of this item. Nevertheless, removal of items based on findings from a single sample should be interpreted cautiously. Future studies should further evaluate the performance of item 2 using additional psychometric approaches such as Rasch analysis, item response theory models, differential item functioning analyses, and qualitative cognitive interviewing to better understand how Arabic-speaking patients interpret this item before considering modification or removal of the item.

The residual correlations observed between items 4 & 21 and items 13 & 18 had substantial implications for model fit. The pair of items 4 (“not doing jobs around the house”) and 21 (“avoiding heavy jobs around the house”) both reflect similar content related to household activity limitations, and their high residual correlation (0.67) indicates shared variance not explained by the general disability factor. Likewise, item 13 (“painful almost all the time”) and item 18 (“sleep less well”) also share a common theme of pain-related disruption, particularly regarding chronic symptoms and sleep disturbance, with a residual correlation of 0.58. These correlations suggest localized dependence between items that go beyond the general latent trait of low-back-related disability. Such residual dependencies have also been documented in previous studies [12,42]. While correlating residuals may be justifiable to improve model fit, it must be done cautiously and based on theoretical rationale. In our case, the paired items clearly share semantic and behavioral overlap, justifying the decision to correlate their error terms. The correlated residuals in the current study were introduced after inspection of modification indices and were therefore post hoc modifications. These residual correlations were added based on both statistical evidence and theoretical overlap between the item contents. We acknowledge that the modified model is theoretically plausible, but the post hoc model requires replication and cross-validation in independent samples before it can be considered stable and generalizable.

In the present study, we employed the WLSMV estimator to conduct CFA of the Arabic RMDQ. This choice was driven by the ordinal nature of the RMDQ items and the violation of multivariate normality, as evidenced by significant skewness and kurtosis. WLSMV is specifically recommended for analyzing models with categorical or ordinal indicators, as it provides robust parameter estimates, corrected standard errors, and accurate model fit indices without assuming normality [28,29,30,31]. This approach contrasts with earlier studies that relied on maximum likelihood (ML) estimation [17] despite the use of ordinal data with limited response categories, which may result in biased parameter estimates, underestimated standard errors, and inaccurate model fit indices. By using WLSMV, our analysis ensured greater accuracy in capturing the true underlying structure of the data, enhancing the credibility of the findings. Moreover, the use of WLSMV aligns with best practices in structural equation modeling for PROMs, as advocated in recent psychometric validation literature [28,29,30,31].

The internal consistency of the Arabic RMDQ in our study was excellent, as reflected by a McDonald’s ω coefficient of 0.91. This surpasses the commonly accepted threshold of 0.70 for adequate internal consistency [35,36], indicating that the items in the Arabic RMDQ are homogeneously related to the underlying construct of back-related disability. McDonald’s ω is preferred over Cronbach’s alpha for estimating internal consistency in CFA contexts, particularly when the tau-equivalence assumption of alpha is not met [36]. While the original validation study of the Modern Standard Arabic RMDQ reported Cronbach’s alpha of 0.73 [25], our use of McDonald’s ω provides a more robust and unbiased estimate of scale reliability. These findings align with previous studies using CFA that have reported strong internal consistency for the RMDQ across different languages and settings [9,17]. It is important to note that these studies reported the scale reliability and internal consistency using Cronbach’s alpha rather than the more appropriate measure that is McDonald’s ω. This high reliability supports the use of the Arabic RMDQ in both clinical and research settings to measure LBP-related disability among Arabic-speaking populations.

A major strength of this study lies in the use of a robust CFA approach to evaluate the structural validity and internal consistency of the Arabic RMDQ. In addition, the study was conducted and reported in accordance with COSMIN guidelines for the evaluation of measurement properties of PROM, including considerations related to study design, statistical analysis, interpretation of structural validity, and reliability assessment. However, this study has some limitations. Although the sample size achieved exceeded the estimated minimum requirement, the sample size remains relatively modest for CFA involving 24 dichotomous indicators and may limit the precision and generalizability of parameter estimates. Measurement invariance across subgroups such as sex, age or symptom duration was not established in the current study. Establishing measurement invariance is paramount to ensure the questionnaire items function in the same way across different clinical characteristics, facilitating valid comparisons among different groups. Therefore, the findings should not yet be interpreted as evidence that the Arabic RMDQ functions equivalently across all Arabic-speaking low back pain populations. The majority of the individuals involved in the present research exhibited chronic LBP. Consequently, prudence must be exercised when extrapolating the outcomes of the current investigation to patients experiencing acute symptoms. The current findings apply most directly to adult Arabic-speaking patients attending outpatient physical therapy clinics in Saudi Arabia, the majority of whom had chronic LBP.

The findings have practical implications for both clinical practice and research. For clinicians, the dominant unidimensional structure of the Arabic RMDQ reinforces its appropriateness as a concise tool for evaluating functional disability in LBP patients. Excellent internal consistency supports its reliability for clinical use. For researchers, the study underscores the importance of using confirmatory approaches and culturally validated instruments when assessing patient-reported outcomes. The current study supports the internal structural validity of the Arabic RMDQ, but additional research is necessary to establish other measurement properties (responsiveness, interpretability, minimal clinically important difference, and measurement error) before making recommendations regarding the use of RMDQ in treatment monitoring and interpretation of clinically meaningful change. Our findings provide a psychometric foundation for further research involving the Arabic RMDQ, including measurement invariance testing, and integration into core outcome sets for LBP studies in the Arab world.

The current study provides supportive evidence for the internal structural validity of the Arabic RMDQ, but additional research is necessary before making stronger claims regarding treatment monitoring and interpretation of clinically meaningful change.

## 5. Conclusions

This study provides supportive evidence for the structural validity and internal consistency of the Arabic version of the RMDQ. The findings provide supportive evidence for a dominant unidimensional structure, but further validation is needed in larger and more diverse samples, including testing of item-level performance and measurement invariance. Given its weak and non-significant loading, future studies should further evaluate item 2 performance using Rasch analysis, item response theory models, and cognitive interviewing with Arabic-speaking patients. The study findings support the appropriateness of the Arabic RMDQ as a valid and reliable tool for assessing LBP-related disability in Arabic-speaking clinical populations with mostly chronic symptoms and support its further use in research and practice.

## Figures and Tables

**Table 1:** Characteristics of participants (N = 113).

Variable	Mean ± SD or *N* (%)
Age (year)	31.03 ± 12.49
Sex	
Male	54 (47.8)
Female	59 (52.2)
Height (m)	1.67 ± 0.10
Mass (kg)	71.54 ± 12.14
Body mass index (kg/m^2^)	25.96 ± 5.61
LBP duration	
Acute (<1 month)	17 (15.04)
Subacute (1–3 months)	19 (16.81)
Chronic (>3 months)	77 (68.14)
RMDQ (0–24)	6.81 ± 4.99

LBP = Low back pain; RMDQ = Roland–Morris disability questionnaire.

**Table 2:** RMDQ CFA parameter estimates.

							95% Confidence Interval of Estimate		
Factor	Indicator	Endorsement Rate	Std. Estimate	Std. Error	z-Value	*p*	Lower	Upper	Threshold
Back-Related Disability	RMDQ1	19.5	0.718	0.087	8.237	<0.001	0.547	0.888	0.861
	RMDQ2	79.6	0.170	0.117	1.455	0.146	−0.059	0.400	−0.829
	RMDQ3	24.8	0.656	0.097	6.788	<0.001	0.467	0.846	0.681
	RMDQ4	32.7	0.700	0.078	8.930	<0.001	0.547	0.854	0.447
	RMDQ5	19.5	0.854	0.068	12.583	<0.001	0.721	0.988	0.861
	RMDQ6	59.3	0.367	0.113	3.253	0.001	0.146	0.588	−0.235
	RMDQ7	20.4	0.620	0.110	5.651	<0.001	0.405	0.836	0.829
	RMDQ8	16.8	0.690	0.083	8.265	<0.001	0.526	0.853	0.962
	RMDQ9	15.0	0.718	0.096	7.472	<0.001	0.530	0.906	1.035
	RMDQ10	46.9	0.651	0.082	7.924	<0.001	0.490	0.812	0.078
	RMDQ11	35.4	0.582	0.091	6.369	<0.001	0.403	0.761	0.375
	RMDQ12	15.9	0.667	0.103	6.457	<0.001	0.465	0.869	0.997
	RMDQ13	31.9	0.691	0.077	8.995	<0.001	0.541	0.842	0.472
	RMDQ14	37.2	0.696	0.079	8.823	<0.001	0.542	0.851	0.327
	RMDQ15	4.4	0.653	0.150	4.346	<0.001	0.358	0.947	1.703
	RMDQ16	19.5	0.686	0.096	7.122	<0.001	0.497	0.874	0.861
	RMDQ17	37.2	0.692	0.087	7.972	<0.001	0.522	0.862	0.327
	RMDQ18	35.4	0.592	0.090	6.566	<0.001	0.416	0.769	0.375
	RMDQ19	1.8	0.570	0.085	6.697	<0.001	0.403	0.737	2.104
	RMDQ20	16.8	0.684	0.107	6.381	<0.001	0.474	0.894	0.962
	RMDQ21	51.3	0.701	0.075	9.350	<0.001	0.554	0.848	−0.033
	RMDQ22	21.2	0.600	0.109	5.481	<0.001	0.385	0.814	0.798
	RMDQ23	21.2	0.848	0.066	12.912	<0.001	0.719	0.977	0.798
	RMDQ24	16.8	0.538	0.126	4.270	<0.001	0.291	0.785	0.962

RMDQ = Roland–Morris disability questionnaire; CFA = Confirmatory factor analysis; std. = Standard.

## Data Availability

The data that support the findings of this study are available from the corresponding author upon reasonable request.
